# Identification of Four Novel *COL4A5* Variants and Detection of Splicing Abnormalities in Three Chinese X-Linked Alport Syndrome Families

**DOI:** 10.3389/fgene.2022.847777

**Published:** 2022-03-17

**Authors:** Sai Wang, Yingfei Shao, Yixiu Wang, Jingru Lu, Leping Shao

**Affiliations:** ^1^ Department of Nephrology, The Affiliated Qingdao Municipal Hospital of Qingdao University, Qingdao, China; ^2^ Department of Dermatology, Peking University First Hospital, Beijing, China; ^3^ Wenzhou Medical University Renji College, Wenzhou, China; ^4^ Darpartment of Hepatic Surgery, Shanghai Cancer Center, Shanghai Medical College, Fudan University, Shanghai, China; ^5^ School of Medicine, Southeast University, Nanjing, China

**Keywords:** x-linked alport syndrome, *COL4A5*, missense mutation, splice site variant, mutation detection

## Abstract

Chronic renal disease associated with X-linked Alport syndrome (XLAS) is relatively rare. However, due to the lack of specificity in the pathologic and clinical manifestations of the disease, it is easy to be misdiagnosed. In this study, we included three Chinese families with XLAS and used targeted NGS to find gene variants. In family X1, the 36-year-old male proband had hematuria, massive proteinuria, sensorineural deafness and ESRD at 33. *In silico* prediction showed the novel c.1424-4C > G variant reduced the score of the normal 3’ splice site from 0.47 to 0.00 (according to BDGP). Transcriptional analysis from his peripheral blood cells indicated that it caused the insertion of an amino acid [p.(Lys474_Gly475insVal)]. In family X2, the proband was a 32-year-old male, who had hematuria, proteinuria, hypertension, hearing loss and progressed into ESRD at 30 years. He carried a novel missense variant c.2777G > T p.(Gly926Val). In family X3, the proband, a 16-year-old male, had hematuria, massive proteinuria, sensorineural deafness and ESRD; the results of renal pathological findings were consistent with AS. He carried a novel variant c.4529-2A > T, so did his mother with ESRD and probable XLAS. Bioinformatic analysis with BDGP showed that it abolished the acceptor site from 0.83 to 0.00. RT-PCR analysis from his kidney tissue indicated that it caused exon 50 skipping and exon 50 skipping along with inserting a cryptic exon derived from intron 49 p.[Gly1510Aspfs*11, Gly1510Alafs*35]. Another novel missense variant c.1552G > A p.(Gly518Arg) was identified in his mother and his aunt. No skewed X-chromosome inactivation was involved in these two female patients. In conclusion, four novel variants in *COL4A5* were identified and transcriptional analysis is essential to investigate the pathogenicity of intronic variants. Thus we found a rare event in a female patient with XLAS caused by two *COL4A5* variants *in trans*.

## Introduction

Alport syndrome (AS) is a rare hereditary glomerulopathy characterized by hematuria, proteinuria, and progressive renal failure, and frequently accompanied by sensorineural deafness and ocular defects, with an incidence of 1/2,320–1/88,866 (Gubler et al., 1981; Gibson et al., 2021). Three inheritance patterns of AS are X-linked (pathogenic mutations in *COL4A5*) (Barker et al., 1990), autosomal recessive (homozygous or compound heterozygous variants in *COL4A3* or *COL4A4*) (Mochizuki et al., 1994) and autosomal dominant (heterozygous *COL4A3* or *COL4A4* variants) (Feingold et al., 1985). Recently, the evidence of digenic inheritance in AS has also been found (heterozygous variants in two different *COL4A3*-*COL4A5* genes) (Mencarelli et al., 2015).

The *COL4A5* gene is located on chromosome Xq22 and encodes the type IV collagen α5 [α5(IV)] chain, the major constituent of the glomerular basement membranes (GBM), cochlea, and eye (Pöschl et al., 2004). Variants in *COL4A5* prevent normal heterotrimer formation of GBM, leading to reduced mechanical stability and splitting of the GBM, as well as other tissue-specific pathological changes (Kruegel et al., 2013). At present, 1,623 different variants in *COL4A5* have been described in LOVD (Leiden Open Variant Database, last update November 2021). In X-linked Alport syndrome (XLAS), 60% of all pathogenic variants are missense, 20% are frameshift mutations, 10% are nonsense mutations, and the remaining 10% are canonical splicing mutations (Savige et al., 2021).

In patients with AS, molecular genetic diagnosis is usually performed by Sanger sequencing of three genes, namely, *COL4A3* (OMIM 120070)*, COL4A4* (OMIM 120131) and *COL4A5* (OMIM 303630). However, these three genes are relatively large, each consisting of 48–53 exons, so conventional Sanger sequencing is laborious, time-consuming, and not a cost-effective method to find candidate mutations. In addition, the diagnosis of XLAS is complicated, which is based on the kidney biopsy, clinical symptoms, extrarenal manifestation, and family history. Nevertheless, ∼10–15% of XLAS cases had a negative family history; those patients harbored *COL4A5 de novo* variants (Lemmink et al., 1997; Haas, 2009; Cervera-Acedo et al., 2017; Sun et al., 2021). Currently, next generation sequencing (NGS) has evolved so much that with little effort and for a reasonable price, a great deal of information can be obtain. Therefore, it is used to diagnose and facilitates variant detection in AS patients.

Genotype-phenotype shows a strong correlation in male XLAS; missense and in-frame variants lead to milder phenotypes, relative to truncating mutations such as nonsense mutations (Jais et al., 2000; Bekheirnia et al., 2010). Horinouchi *et al.* showed that male patients with XLAS caused by aberrant splicing exhibit different prognoses, depending on whether their mutations are truncating or non-truncating transcripts (Horinouchi et al., 2018). To most accurately predict kidney prognosis, it is essential to perform transcriptional analysis when detecting mutations including splice site and point variants. *In silico* analysis tools have been used to predict transcriptional effects of variants such as the Human Splicing Finder and Berkeley Drosophila Genome Project (BDGP). However, it is very difficult to make a reliable prediction of splicing patterns without transcriptional analysis. The ideal experimental method to identify splicing alterations is to analyze RNA expression profile of target tissues from patients. However, in many cases, this type of sample is not always available from the patient, or it has been obtained in ways that cannot ensure its stability. In addition, *in vitro* splicing assay with minigene has also been used to detect aberrant splicing caused by variants in *COL4A5* (Malone et al., 2017; Horinouchi et al., 2018; Horinouchi et al., 2019; Gao et al., 2020). Currently, the best approach to identify splicing variants with a role in disease is to use a combination of these *in vivo*, *in silico* and *in vitro* analyses.

In this study, we used targeted NGS to identify two missense and two splicing variants in three Chinese families with XLAS. Then, we further analyzed the consequences of these gene variants at mRNA level using *in silico* and *in vivo* approaches.

## Materials and Methods

### Ethical Compliance

This study and procedures were approved by the Ethics Committee of the Affiliated Hospital of Qingdao University. Informed written consent was obtained from all participants prior to participating in the study.

### Subjects

Ten Chinese patients with suspected AS from three non-consanguineous families were included in this study. AS was clinically diagnosed if the proband and other family members between them met at least three of the following criteria (Flinter et al., 1988): 1) positive family history of hematuria with or without chronic renal failure; 2) electron microscope evidence of AS in renal biopsy specimens; 3) characteristic ophthalmic signs; 4) high-tone sensorineural deafness.

### Clinical Investigations

Clinical data of three families with AS, including renal and extrarenal manifestations, GBM ultrastructural changes, immunohistochemical changes of α(IV) chains in renal and/or skin tissues, and family history, were collected in this study. Besides, all subjects with hematuria underwent microscopic examination of the urinary sediment to confirm the presence of red blood cells at the Affiliated Qingdao Municipal Hospital of Qingdao University. The urinary dysmorphic red blood cells or cast formation demonstrated that the hematuria was the origin of glomeruli.

### Culture of Skin Fibroblasts

Skin tissue specimens (5 mm in diameter; 1 mm in depth) were obtained from the forearms of three patients and healthy subjects (family X3: II-1, II-3 and III-2). Skin specimens were washed in 0.01 M phosphate buffer and DMEM, carefully removing epithelium and fat. Then the cells were cut into 1–2 mm fragments and inoculated into cell culture bottles containing DMEM (10% fetal bovine serum, L-glutamine (20 mM), penicillin (100 U/mL), streptomycin (100 μg/ml)) and incubated at 37°C incubator (containing 5% CO_2_).

### Targeted NGS and Bioinformatics Analysis

Genomic DNA was extracted from peripheral blood leukocytes by GenElute blood genomic DNA extraction kit (Sigma, NA 2010). Targeted NGS was performed on the Hiseq2500 platform (Illumina) by BGI (Tianjin, China) as paired-end 100-bp reads (PE100). The gene panel consists of 4,811 genes, including *COL4A3*, *COL4A4* and *COL4A5*, and was designed to capture coding regions, splice sites, and the immediately adjacent intronic sequences. Raw data processing was performed using the Illumina Pipeline (version 1.3.4). Empty reads, adaptor sequences, and low-quality sequences (reads that contained >10% Ns in the read length, 50% reads with a quality value of <5 and with an average quality of <10) were removed from the raw data. The remaining sequences were called clean reads for further analysis. Clean reads that passed were then aligned to the human reference genome (UCSC hg19) using the Burrows Wheeler Aligner software (University of California, Santa Cruz, CA, USA). Small insertion/deletions (indels) were detected by SAM tools Pileup software (http://sourceforge.net/projects/samtools/), and single nucleotide variants (SNVs) were identified though SOAPsnp software (http://soap.genomics.net/projects/biobwa/). The variant calling file (VCF) containing these variants was annotated with 1,000 genomes, the Single Nucleotide Polymorphism Database (dbSNP), Exome Sequencing Project (ESP) and the Exome Aggregation Consortium (ExAC). After the selection process, we use the online software (SIFT, PolyPhen-2, and MutationTaster) to predict the pathogenicity of putative missense mutations. Moreover, potential splicing variants were analyzed by BDGP.

### Sanger Sequencing

The suspected candidate variant sites and its flanking regions were amplified by PCR and were validated by Sanger sequencing using an ABI Prism 3700 DNA Analyzer (Applied Biosystems, CA, USA). When heterozygous deletion or insertion variants were suspected, the PCR products were subcloned into the PGEM-T Easy plasmid (Promega, A1360) and sequenced using the T7/SP6 primers.

### Splicing Analysis

Total RNA was extracted from peripheral blood cells (family X1: I-1, II-2, II-3, III-1 and III-2), renal tissues (renal biopsy tissue of family X3: III-2 and a control taken from one nephrectomy specimen with renal cell carcinoma and obtained from unaffected areas in that kidney) and cultured skin fibroblasts (family X3: II-1, II-3 and III-2) with the TRIzol reagent (Invitrogen, Carlsbad, CA). First-strand cDNA synthesis was carried out through random-primed reverse transcription using the PrimeScript RT reagent Kit with gDNA Eraser (Takara, Japan) according to the manufacturer’s instruction (Wang et al., 2020). The resulting cDNA was amplified by PCR using specific primers containing variant sites. The PCR amplification reaction were performed in a 20 μL volume with the first denaturation step at 95°C for 30 s, subsequently followed by 29 cycles with denaturation at 95°C for 30 s, annealing at 62–53°C for 30 s and elongation at 72°C for 2 min, and final extension at 72°C for 5 min. The PCR products were separated by 1.5% agarose gel electrophoresis and each band signal was analyzed by sequencing.

### X-Chromosome Inactivation Analysis

To indirectly access the X-chromosome inactivation patterns of two female patients in family X3, the cDNAs of cultured skin fibroblasts were amplified for 25 cycles and inserted into pGEM-T Easy plasmid. Then, the vector plasmids with cloned insert were transformed into DH5α competent *E. coli* cells and multiplied in the Luria-Bertani (LB) broth and spread evenly on the IPTG/x-GAL (Invitrogen, United States) coated ampicillin-LB agar plates for 16 h at 37°C. Forty-eight collected monoclonal colonies were picked up and extracted using PurePlasmid Mini Kit (Cwbio, China). The plasmid DNA was sequenced using T7/SP6 primers. Sequence analysis and alignment were performed using Chromas 2.31 and Vector NTI Advance 10.

### Application of 2015 ACMG Guidelines

The ACMG guidelines were applied to all variants identified in this study, and final classification of pathogenic, likely pathogenic, variants of uncertain significance, likely benign, or benign was assigned according to the published algorithm (Richards et al., 2015).

## Results

### Clinical Characteristics

**FIGURE 1:**
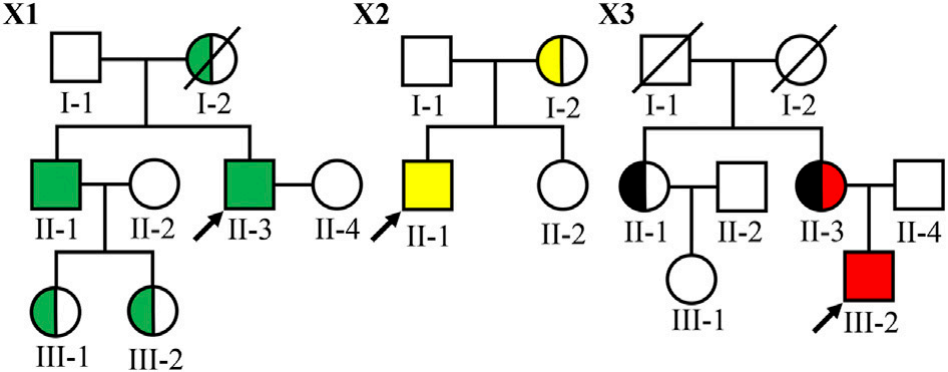
Pedigree of three Chinese families with XLAS. Filled symbols represent affected individuals (green: the *COL4A5* c.1424–4C > G variant carriers; yellow: the *COL4A5* c.2777G > T p.(Gly926Val) variant carriers; black: the *COL4A5* c.1552G > A p.(Gly518Arg) variant carriers and red: the *COL4A5* c.4298–2A > T variant carrier). The symbol with a slash corresponds to a decreased individual. Arrow indicates the proband.

Three families with AS were collected in this study (Figure 1) The clinical manifestations and laboratory findings of nine Chinese patients with AS are summarized in Table 1.

**TABLE 1:** Clinical manifestations of affected individuals in families X1-X3.

				Urinalysis				Renal biopsy			Skin biopsy	
Family	Affected member/gender/age^a^	Blood pressure (mmHg)	eGFR (mL/min/1.73 m^2^)	Glomerular hematuria	Proteinuria (g/24 h)	Ocular	Sensorineural	LM	EM	α3(IV)/α5(IV)chain	α5(IV) chain	ESRD/age^b^
					(0–0.03)	Abnormality	Deafness					
X1	II-1/M/38y	168/105	4.5	+	6.32	−	+	ND	ND	ND/ND	ND	32y
	II-3/M/36y	145/102	19.3	+	6.05	−	+	ND	ND	ND/ND	ND	33y
	III-1/M/10y	120/75	90.5	+	0.00	−	−	ND	ND	ND/ND	ND	No
	III-2/M/8y	115/85	83.9	+	0.00	−	−	ND	ND	ND/ND	ND	No
X2	I-2/F/56y	128/85	67.2	+	2.50	−	−	ND	ND	ND/ND	ND	No
	II-1/M/32y	169/118	13.8	+	5.97	−	+	ND	ND	ND/ND	ND	30y
X3	II-1/F/50y	110/70	102.4	+	0.00	−	−	ND	ND	ND/ND	+	No
	II-3/F/48y	165/90	3.9	+	6.35	−	+	ND	ND	ND/ND	−	29y
	III-2/M/16y	170/100	31.4	+	5.67	−	+	Glomerular scarring 25%, foam cells	Typical Alport finding	−/−	−	16y

a Age at the first admission; eGFR, estimated glomerular filtration rate (the normal reference range of people aged 18 years or older: >90 ml/min/1.73 m^2^; under 18 years old: 89–165 ml/min/1.73 m^2^); LM, light microscopy; EM, elecron microscopy; ESRD, end-stage renal disease.

b Age at diagnosis; F, female; M, male; ND, not done; -, negative; GBM, glomerular basement membrane.

In family X1, the proband II-3, male, was 36 years old at the time of genetic analysis. Hematuria and proteinuria had been noted for the patient from 3 years of age. His conditions progressed to ESRD at 33 years. He also had hypertension and hearing loss. The proband’s older brother had been diagnosed with hematuria and proteinuria at the age of 2 and developed into ESRD at the age of 32. He also had hearing loss and no ocular abnormality. His eldest and younger sons had hematuria at the age of 3 and 4, respectively. The proband’s mother died of ESRD.

In family X2, the proband II-1 was a 32-year-old male at the time of genetic analysis. He had hematuria from childhood and proteinuria at 5 years of age. At the age of 20, he was diagnosed with hypertension and sensorineural hearing loss, but no with ocular abnormality. The conditions progressed to ESRD at 30 years and currently, his estimated glomerular filtration rate (eGFR) was 13.8 ml/min/1.73 m^2^. His mother had hematuria, and proteinuria and her eGFR was 67.2 ml/min/1.73 m^2^ at 56 years of age. She had neither ocular abnormalities nor hearing loss.

In family X3, the proband III-2 was a 16-year-old boy at the time of genetic analysis. He had hematuria and proteinuria from 3 years old, hypertension, moderate to severe sensorineural hearing loss and ESRD. At the age of 15, kidney biopsy was performed. Electron microscopy demonstrated irregular thin and thickened areas with splintered and irregular multi-laminated appearance of the lamina densa, as well as the changes with basket-weave pattern in the thickened areas. The α3 and α5 collagen IV chains were absent in renal glomerulus and tubules. His mother (currently 48-year-old) also had persistent hematuria, proteinuria, hypertension, and sensorineural hearing loss, while the onset time was not clear. Her conditions developed into ESRD at 29 years old and received dialysis from 39 years of age. His aunt (currently 50-year-old) had isolated microhematuria from 20 years of age, but renal function and hearing were normal. The proband (III-2), the proband’s aunt (II-1), and the proband’s mother (II-3) underwent skin biopsies. Among of them, two of them (II-3 and III-2) presented negative for α5(IV) chains in the immuno-staining test of epidermal basement membrane. The proband’s grandfather and grandmother died without a definite cause at the age of 45 and 60, respectively.

### Identification of Four Novel Variants in *COL4A5*

To make a definite diagnosis and find pathogenic gene variants, targeted NGS was performed. Four novel variants in *COL4A5* gene were identified in ten patients with AS, including 2 missense [c.2777G > T p.(Gly926Val) and c.1552G > A p.(Gly518Arg)], and 2 intron variants (c.1424-4C > G and c.4529–2A > T) (Table 2), and confirmed by Sanger sequencing. An allele of *COL4A5* gene was detected variants inherited from the mother in each of the three probands. Among 10 affected individuals, four of them were hemizygotes and five were heterozygotes. In family X3, the proband’s mother (II-3) carried two variants (c.1552G > A and c.4529-2A > T), whereas the proband was hemizygote and only carried the variant c.1552G > A (Figure 1). Therefore, both variants located on two different *COL4A5* alleles (*in trans*) and the proband’s mother was a compound heterozygote. Obviously, the variant c.1552G > A in patients II-1 and II-3 inherited from their parents I-1 or I-2. Due to the lack of genetic information of I-1 and I-2, it is unlikely to confirm the origin of c.4529–2A > T.

**TABLE 2:** Classification of the variants identified in this study.

						*In silico* analysis		
Family	Gene	Site	Nucleotide change	RNA change	Protein change	SIFT	PolyPhen-2	MutationTaster	1000g	ExAc	Classification	Evidence
X1	*COL4A5*	Intron 21	c.1424–4C > G	r.1423_1424insuag	p.(Lys474_Gly475insVal)	NA	NA	NA	0	0	Pathogenic	PS3+PM2+PM4+PP3+PP4
X2	*COL4A5*	Exon 33	c.2777G > T	r.(?)	p.(Gly926Val)	D	PD	DC	0	0	Likely Pathogenic	PM1+PM2+PP3+PP4
X3	*COL4A5*	Intron 49	c.4529–2A > T	r.[4529_4706del, 4529_4706delins4529-1737_4529-1586]	p.[Gly1510Aspfs*11, Gly1510Alafs*35]	NA	NA	NA	0	0	Pathogenic	PVS1+PM2+PP3+PP4
		Exon 23	c.1552G > A	r.1552g>a	p.(Gly518Arg)	D	PD	DC	0	0	Likely Pathogenic	PM1+PM2+PP3+PP4

D, damaging; PD, probably damaging; DC, disease causing; T, tolerated; P, Polymorphism; NA, not applicable; 1000g, Genomes Project; ExAc, Exome Aggregation Consortium; gnomAD, genome aggregation database; PM, pathogenic moderate; PP, pathogenic supporting; PS, pathogenic strong; PVS, pathogenic very strong.

### Two Variants Altered the Splicing Process

**FIGURE 2:**
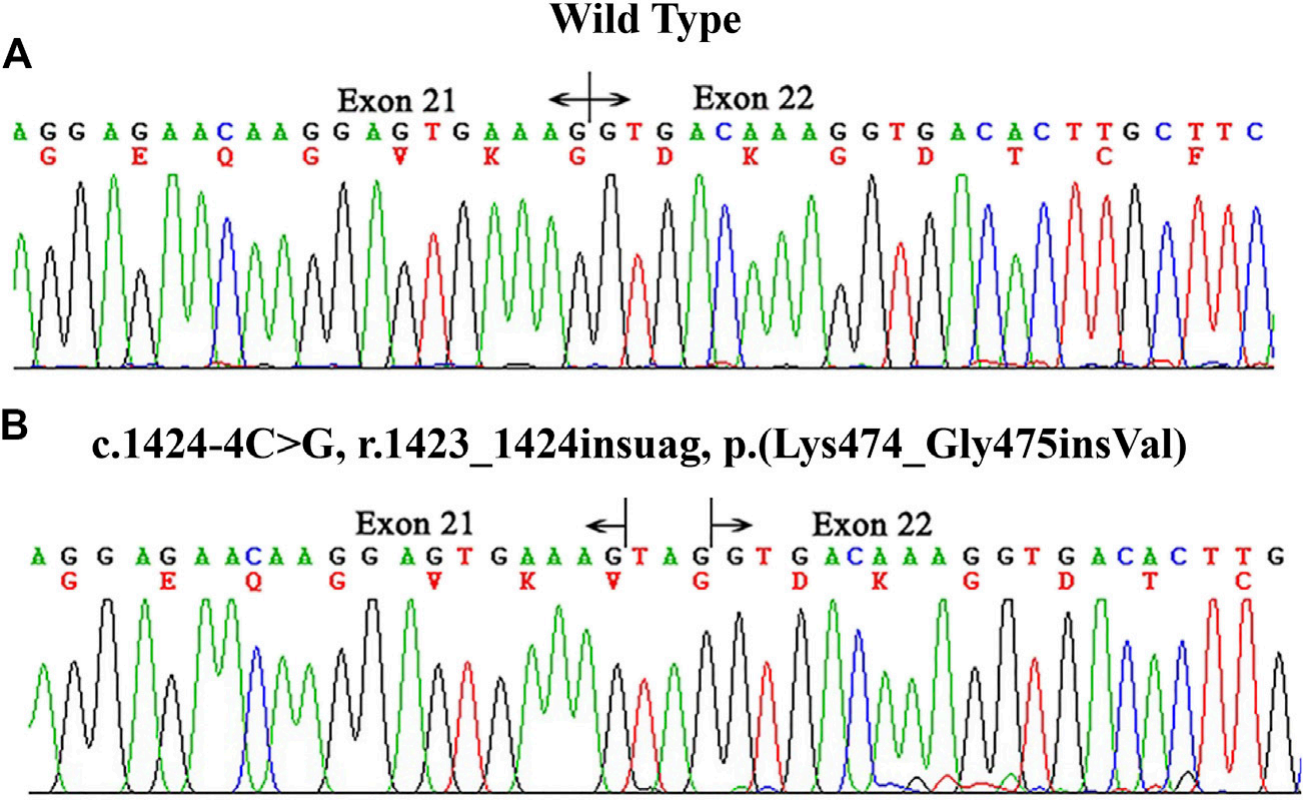
cDNA sequencing results of *COL4A5* in the proband of family X1. **(A)** the wild type. **(B)** the proband (II-3). The hemizygous variant c.1424–4C > G p.(Lys474_Gly475insVal) altered the 3′ splice site of exon 22 and resulted in an insertion of three nucleotides between exons 21 and 22.

The variant c.1424–4C > G affected the G nucleotide at position -4 of exon 22. Analysis of this substitution with BDGP showed that it marginally decreased the score of the normal 3′ splice site from 0.47 to 0.00. To examine whether this variant affected mRNA splicing, we extracted RNA from the patients’ peripheral blood cells in family X1. cDNA sequencing results showed that the variant resulted in insertion of three nucleotides from the 3’ end of intron 21 (r.1423_1424insuag) between the exon 21 and 22 and did not alter the open reading frame [p.(Lys474_Gly475insVal)] (Figure 2). The COL4A5 protein encoded by this altered mRNA would insert an amino acid (valine) in Gly-X-Y repeats collagen region.

**FIGURE 3:**
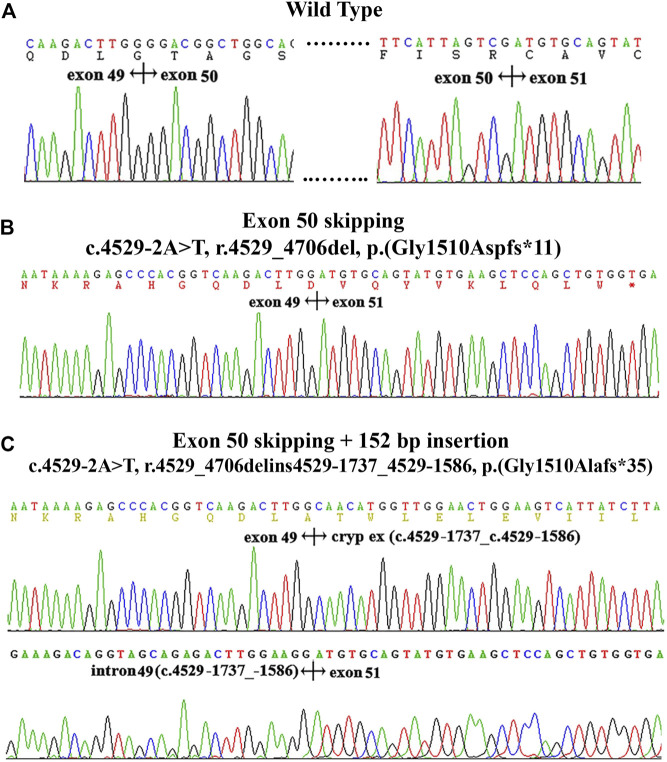
cDNA sequencing results and electropherogram of kidney tissues from patients in family X3. **(A)**: the wild type. **(B)**: the skipping of exon 50. **(C)** the skipping of exon 50 and insertion of a 152 bp fragment from a cryptic exon of intron 49 (c.4529-1737_529-1586).

The variant c.4529-2A > T located at intron 49, as the canonical splice acceptor site, may abolish the acceptor site predicted by BDGP (mutant score: 0.00 compared to the wild-type score: 0.83). To determine the effect of this variant on the transcript level, we extracted RNA from proband’s kidney tissue in family X3. RT-PCR analysis showed that the normal control generated a unique product (872 bp), while the mutant produced two different bands (700 bp and 852 bp, respectively). Sequencing analysis of all bands indicated that the 872 bp product from the normal control was a transcript containing exon 50 (Figure 3A). The smaller splice of 700 bp was a transcript missing exon 50 (Figure 3B), while the larger fragment of 852 bp was a transcript without exon 50 but containing a 152 bp cryptic exon (c.4529-1737_c.4529-1586) from intron 49 (Figure 3C). And approximately 40% (19/48) of cDNA products from proband’s renal tissue inserted into the pGEMT Easy vector lacked the whole exon 50, 60% (29/48) lacked exon 50 with insertion of a cryptic exon.

The variant c.1552G > A is located at position 36 in exon 23. In previous studies (Han et al., 2019; Wang et al., 2020), we have found exonic variants can also cause abnormal splicing and these variants should be categorized as splicing variants. Therefore, in order to analyze whether this variant affects splicing process, we also extracted RNA from cultured skin fibroblasts of three patients’ (II-1 and II-3) from family X3 for sequencing. The results showed this variant did not alter splicing process (Supplementary Figure S1). However, since RNA from patients is unavailable in family X2, we did not analyze the effect of the variant c.2777G > T p.(Gly926Val) on transcription.

### A Skewed X-Inactivation Pattern was not Involved in Two Female Patients of Family X3

Women with heterozygous mutation in *COL4A5* have a wide variability in outcomes (Jais et al., 2003), the explanation for it remains unclear, but is determined at least in part by X-chromosome inactivation (Rheault et al., 2010). Therefore, in order to indirectly verify whether a skewed X-inactivation pattern exited in both female patients in family X3, we analyzed mRNA expression level of the variants c.1552G > A and c.4529–2A > T in their cultured skin fibroblasts. The results of T-A cloning indicated 56% (27/48) of cDNA products were the c.1552G > A mutant alleles and 44% (21/48) were normal alleles in patient II-1. Similarly, in patient II-3, 60% (29/48) were the c.1552G > A mutant alleles and 40% (19/48) were not them. In addition, we also analyzed the transcription products of c.4529–2A > T in fibroblasts of the patient II-3 using another pair of primers covering exon 50 and flanking sequences. The cDNA products missing exon 50 and missing exon 50 with insertion of a cryptic exon were 53% (10/19) and 47% (9/19), respectively, which was similar to those in the kidney. Therefore, X-inactivation analysis results showed a balanced inactivation of both *COL4A5* alleles in patients II-1 and II-3.

### ACMG Classification

As shown in Table 2, according to the 2015 ACMG standards and guidelines, as well as RNA analysis results, the splice site variant c.4529-2A > T p.[Gly1510Aspfs*11, Gly1510Alafs*35] can be classified as “pathogenic” due to its null effect. The variant c.1424-4C > G p.(Lys474_Gly475insVal) causes the in-frame insertion (insertion of one amino acid) confirmed by *in vivo* studies that disrupts the leitmotif of Gly-X-Y, so it can also be classified as “pathogenic”. Both missense variants affected glycine in the collagenous domain of the α5(IV) chain, which is the most common and well-known amino acid substitution resulting in a disruption of collagen helix and therefore can be classified as “likely pathogenic”.

## Discussion

**FIGURE 4:**
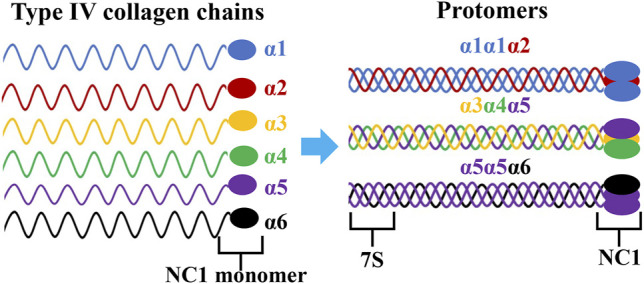
Triple helical organization of the type IV collagen family. Six genetically different type IV collagen α-chains (α1-α6) are arranged into three triple helical protomers that differ in their chain composition. All promoters contain a 7S triple helical domain at the N-terminal that is rich in cysteine and lysine residues, a central collagenous triple helix domain, and a non-collagenous (NC1) trimer at the C- terminal.

AS is a rare and monogenic nephropathy that generally starts with hematuria and proteinuria, which often progresses to ESRD (Pirson, 1999). Type IV collagen is an essential structural component of the mammalian basement membrane and consists of six distinct α chains, α1-α6 (IV), encoded by *COL4A1-COL4A6* genes, respectively. Each of these genes have a common primary structure which contains an amino-terminal 7S domain, a collagen domain with Gly-X-Y repeats, and a carboxy-terminal NC1 domain (Zhou et al., 1994; Hudson et al., 2003; Cosgrove and Liu, 2017). There are three collagen IV heterotrimers, α1α1α2(IV), α3α4α5(IV) and α5α5α6(IV). These trimers form triple-helical molecules called protomers *via* specific NC1 domain interactions that further assemble into the basement membrane superstructure through NC1 and 7S domain interactions (Figure 4) (Timpl et al., 1981; Zhou et al., 1994; Boutaud et al., 2000). The repetitive glycine residue in the collagenous domain is essential for proper assembly of the collagen triple helix and the amino-acid residues in the X-Y positions are located on the outside of the triple helix (Kawai et al., 1996). According to previous results, almost missense variants in AS patients, apart from conserved amino acids in the NC1 domain, were glycine substitutions in the collagenous domain. These variants can lead to distortion of the collagen triple helix during folding and molding.

Variants in *COL4A5* caused abnormal α5(IV) expression, with a typically complete absence of α5(IV) in GBM and Bowman’s capsule in men and a mosaic expression pattern in women (Kashtan, 1998). Nowadays, the genotype-phenotype correlation in male XLAS is relatively well established; missense or in-frame variants show milder phenotypes than truncating mutations (Jais et al., 2000; Bekheirnia et al., 2010; Chen et al., 2021). Regarding splicing mutations, Horinouchi *et al.* analyzed 14 XLAS families with variants in atypical splicing patterns, revealing that the median age at ESRD onset was 20 years for those with truncating variants, and 29 years for those with non-truncating variants (Horinouchi et al., 2018). Recently, splicing modulation of exon skipping by antisense oligonucleotide (ASO) has been shown to be useful in cases with truncating variants. ASOs have been successfully used to treat a variety of genetic disorders, such as Eteplirsen for the treatment of Duchenne muscular dystrophy, with the aim of converting truncating variants into exon skipping that may cause non-truncating variants at the transcription level. Similarly, Yamamura *et al.* have developed an exon-skipping therapeutic strategy that prevents progression of kidney failure in a *Col4a5* mutant mouse (Yamamura et al., 2020), which may be available in future for patients with truncating *COL4A5* mutations. Therefore, in XLAS, it is necessary to understand the differences between splicing transcripts to assess renal prognosis, conduct genetic counseling, and develop further treatment strategies.

In this study, a total of 4 novel variants of *COL4A5* gene were identified, including two missense [c.2777G > T p.(Gly926Val) and c.1552G > A p.(Gly518Arg)] and two splicing variants (c.1424–4C > G and c.4529–2A > T), which expand the mutation spectrum of *COL4A5* gene in LOVD. As for the two novel missense mutations, the assessment results using 2015 ACMG standards and guidelines suggested likely pathogenic. The novel intronic variant c.1424-4C > G p.(Lys474_Gly475insVal) in this study inserted one amino acid between exon 21 and exon 22 by RT-PCR analysis of RNA products extracted from patients’ peripheral blood lymphocytes (family X1: II-1, II-3, III-1 and III-2), which interrupts continuous Gly-X-Y triplets. We observed full segregation of above variants in the respective families.

Another splicing variant c.4529–2A > T led to two different transcriptional products using the proband’ renal tissues. One aberrant splicing pattern was the skipping of exon 50, which affected the reading frame downstream of residue 1,510 and created a premature stop signal of translation at residue 1,520 [p.(Gly1510Aspfs*11)]. Another one was the skipping of exon 50 with insertion of a cryptic exon of 152 bp derived from intron 49, which resulted in a frame shift starting with codon 1,520 and a premature termination of translation at codon 1,544 [p.(Gly1510Alafs*35)]. As a canonical splice site, the substitution of adenine for thymine significantly decreased the recognition of splice acceptor site. Of note, this variant also prevents the normal splicing process of intron 49 and activates a potential exon located in the deep intron 49. Universally, these potential exons were used by forming a consensus sequence (5′AG or 3′GT) of new splice sites (donor or acceptor site) or by generating more powerful exon splice enhancers to enhance the pre-existing splice sites. However, further sequencing of intron 49 did not find any suspicious variants. We suspect that this variant may alter the secondary structure of RNA.

In family X1, both male patients II-1 and II-3 had the splicing variant c.1424-4C > G p.(Lys474_Gly475insVal), and they developed ESRD at the age of 32 and 33, respectively. The male proband (III-2) in family X3 carried the hemizygous variant c.4529-2A > T p.[Gly1510Aspfs*11, Gly1510Alafs*35] and progressed to ESRD at the age of 16. Compared with the variant c.4529-2A > T carriers, the reason for the later onset of ESRD in the variant c.1424-4C > G carriers is that c.1424–4C > G produces a non-truncated protein product based on RNA splicing analysis, which is consistent with previous studies (Hashimura et al., 2014; Horinouchi et al., 2018).

Hemizygous male patients are usually severely affected, while female heterozygotes have a broad spectrum of clinical manifestations from isolated hematuria to ESRD (Migeon, 2008; Temme et al., 2012). *Jais et al.* reported that 12% of female XLAS patients progress to ESRD presumably before 40 years, which may be related to the random X-chromosome inactivation and allelic heterogeneity (Jais et al., 2003). Though targeted NGS and co-segregation analysis in family X3, the patient II-3 had two variants c.1552G > A p.(Gly518Arg) and c.4529-2A > T p.[Gly1510Aspfs*11, Gly1510Alafs*35] located on two different X-chromosomes. RNA analysis of this patient’s skin fibroblasts showed that the transcripts from two mutant alleles were detected at a compatible high abundance (60 *vs*. 40%), which indicated that a skewed X-inactivation pattern was not present in this patient. Additionally, this patient progressed to ESRD at the age of 29, which may be related to two potential pathogenic variants *in trans*. Of note, the severe symptoms in female patients caused by compound heterozygous *COL4A5* variants are extremely rare.

In summary, we identified four novel *COL4A5* variants in three families, including two missense and two splicing variants with targeted NGS. And the two splicing variants were confirmed to alter mRNA splicing by RT-PCR assay. Our findings expand the spectrum of variants in *COL4A5* and strengthen our understanding of genotype-phenotype correlations in XLAS. In addition, we described a rare event in a female patient with XLAS caused by a compound heterozygous *COL4A5* genotype. Detecting variants in affected family members is also important for prenatal diagnosis and subsequent diagnosis of their offspring, thus reducing the need for more invasive renal biopsies.

## Data Availability

The datasets for this article are not publicly available due to concerns regarding participant/patient anonymity. Requests to access the datasets should be directed to the corresponding author.
