# Supplementary figures and images for: Identification of Four Novel COL4A5 Variants and Detection of Splicing Abnormalities in Three Chinese X-Linked Alport Syndrome Families
Source: Front Genet. 2022 Mar 17;13:847777. doi: 10.3389/fgene.2022.847777 (PMC8968133; doi:10.3389/fgene.2022.847777)

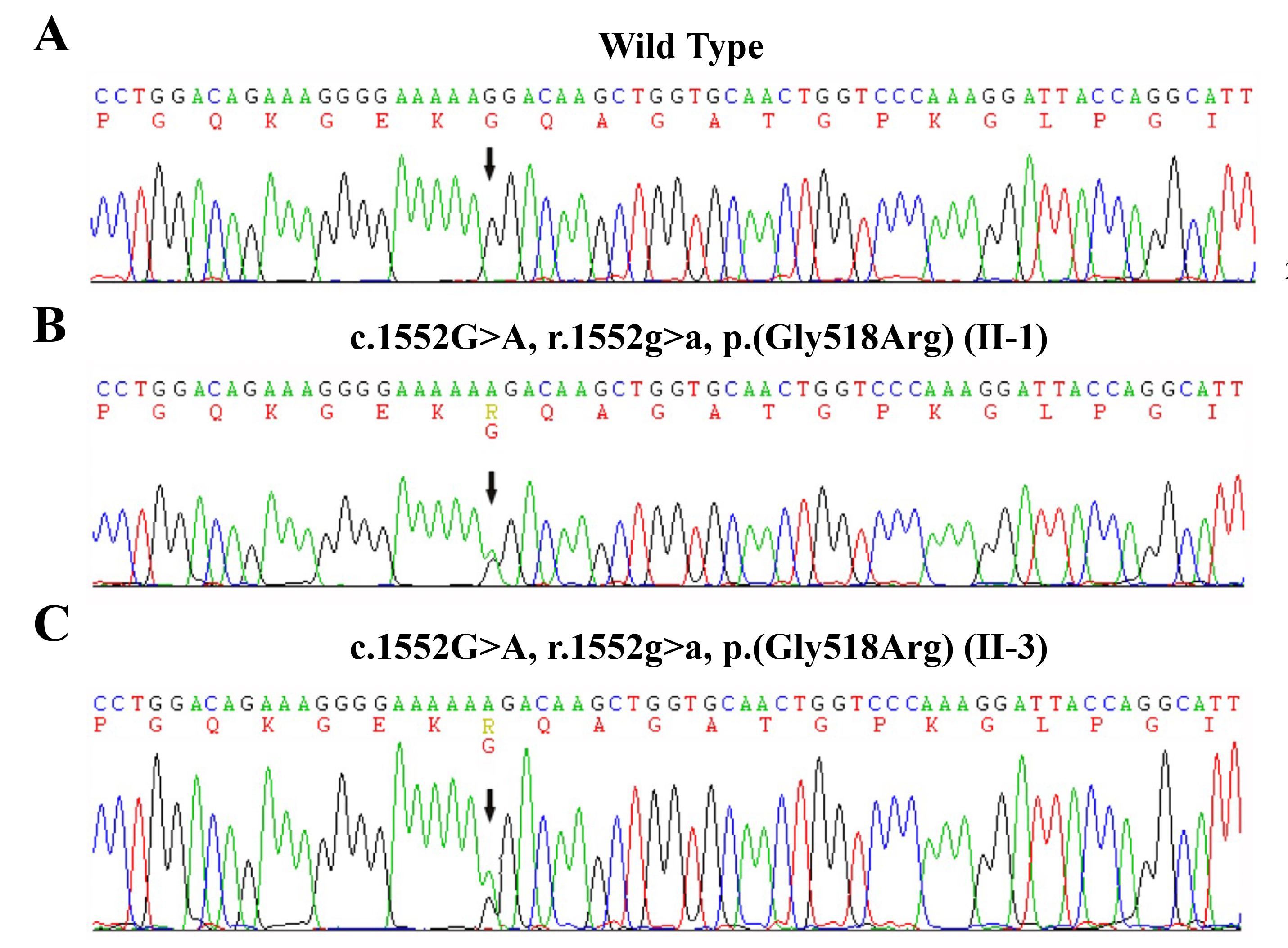

Supplement: Supplementary file 1 [file Image1.TIF]
